# Inhibition of OGG1 ameliorates pulmonary fibrosis via preventing M2 macrophage polarization and activating PINK1-mediated mitophagy

**DOI:** 10.1186/s10020-024-00843-6

**Published:** 2024-05-31

**Authors:** Wenjuan Wu, Hongxia Jia, Song Chen, Xinran Ma, Shuai Zhou, Lingxiao Qiu, Xinhui Wu, Ping Li, Heying Chu, Guojun Zhang

**Affiliations:** 1 Department of Respiratory and Critical Care Medicine, The First Affiliated Hospital of Zhengzhou University, 1 Jianshe East Road, Zhengzhou, 450000 Henan China; 2 Department of Geriatric Medicine, Henan Provincial People’s Hospital, Zhengzhou University, Zhengzhou, 450000 China; 3 Translational Research Institute, Henan Provincial People’s Hospital, Academy of Medical Science, Zhengzhou University, Zhengzhou, China; 4 Department of Respiratory and Critical Care Medicine, the Second Affiliated Hospital of Army Medical University, Chongqing, 400037 China; 5 Department of Traditional Chinese Medicine, Zhengzhou Shuqing Medical College, Zhengzhou, 450064 China

**Keywords:** Pulmonary fibrosis, OGG1, PINK1/parkin, Macrophages, Mitophagy

## Abstract

**Background:**

8-Oxoguanine DNA glycosylase (OGG1), a well-known DNA repair enzyme, has been demonstrated to promote lung fibrosis, while the specific regulatory mechanism of OGG1 during pulmonary fibrosis remains unclarified.

**Methods:**

A bleomycin (BLM)-induced mouse pulmonary fibrosis model was established, and TH5487 (the small molecule OGG1 inhibitor) and Mitochondrial division inhibitor 1 (Mdivi-1) were used for administration. Histopathological injury of the lung tissues was assessed. The profibrotic factors and oxidative stress-related factors were examined using the commercial kits. Western blot was used to examine protein expression and immunofluorescence analysis was conducted to assess macrophages polarization and autophagy. The conditional medium from M2 macrophages was harvested and added to HFL-1 cells for culture to simulate the immune microenvironment around fibroblasts during pulmonary fibrosis. Subsequently, the loss- and gain-of function experiments were conducted to further confirm the molecular mechanism of OGG1/PINK1.

**Results:**

In BLM-induced pulmonary fibrosis, OGG1 was upregulated while PINK1/Parkin was downregulated. Macrophages were activated and polarized to M2 phenotype. TH5487 administration effectively mitigated pulmonary fibrosis, M2 macrophage polarization, oxidative stress and mitochondrial dysfunction while promoted PINK1/Parkin-mediated mitophagy in lung tissues of BLM-induced mice, which was partly hindered by Mdivi-1. PINK1 overexpression restricted M2 macrophages-induced oxidative stress, mitochondrial dysfunction and mitophagy inactivation in lung fibroblast cells, and OGG1 knockdown could promote PINK1/Parkin expression and alleviate M2 macrophages-induced mitochondrial dysfunction in HFL-1 cells.

**Conclusion:**

OGG1 inhibition protects against pulmonary fibrosis, which is partly via activating PINK1/Parkin-mediated mitophagy and retarding M2 macrophage polarization, providing a therapeutic target for pulmonary fibrosis.

## Background

Pulmonary fibrosis is a chronic and progressive interstitial pneumonia, characterized by alveolar structure destruction, extracellular matrix remodeling and fibroblast proliferation, leading to irreversible distortion of the lung’s architecture and functional failure (Martinez et al. 2017). Currently, nintedanib and pirfenidone are the only two FDA-approved drugs for clinical application of patients with pulmonary fibrosis, which present a modest benefit at retarding lung function decline within one year; however, these two medications can not cure this disease, and up to date, there are no effective therapies to cure pulmonary fibrosis completely (Fleetwood, et al. 2017). Therefore, the better understanding of the basic molecular mechanism may be beneficial to discover therapeutic modalities.

Mitochondrial dysfunction has emerged as a critical contributor to a variety of diseases, including cancer, neurodegenerative, cardiovascular and lung diseases (Ajoolabady et al. 2022; Sharma et al. 2021; Mishra et al. 2021). Mitophagy, a selective mitochondrial autophagy, is triggered by excessive oxidative stress attributed to elevated mitochondrial-derived ROS, and exerts a crucial function in eliminating damaged mitochondria, ultimately contributing to an eventual turnover of dysfunctional mitochondria and thereby maintaining mitochondrial homeostasis (Youle and Narendra 2011). It is well known that PTEN induced kinase 1 (PINK1) and Parkin are essential for the maintenance of mitochondrial quality through mitophagy activation, and PINK1/Parkin-dependent mitophagy has been identified as a potential target for the treatment of pulmonary fibrosis (Bueno et al. 2015; Liu et al. 2020).

8-Oxoguanine DNA glycosylase (OGG1) is a well-known DNA repair enzyme that removes 8‐oxoG from DNA duplexes during the process of base excision repair and is involved in the repair of oxidation‐induced damage of DNA. Recent studies have indicated that OGG1 inhibits the activation of PINK1/Parkin mitophagy pathway and thereby aggravates mitochondrial functional damage (Zhao et al. 2023). Of note, OGG1 can increase α-SMA level through promoting α-SMA polymerization into stress fibers (Luo et al. 2014). OGG1 is also demonstrated to promote lung fibrosis via regulating TGFβ/SMAD signaling and activating fibroblasts (Wang et al. 2020; Song et al. 2023). Therefore, OGG1 has been considered as a critical mediator in the pathogenesis of pulmonary fibrosis. However, the molecular mechanism of OGG1 against pulmonary fibrosis has been not elucidated completely. Considering above, whether OGG1-related PINK1/Parkin mitophagy is involved in this regulatory process is deserved to be explored.

Therefore, in the present study, we focused on the regulatory mechanism of OGG1 in pulmonary fibrosis using both in vivo and in vitro approaches. We set up a bleomycin (BLM)-induced mouse pulmonary fibrosis model, and demonstrated that TH5487, the small molecule OGG1 inhibitor, could attenuate pulmonary fibrosis, activate PINK1/Parkin mitophagy and reduce M2 macrophage polarization. Subsequently, the loss- and gain-of function experiments were conducted in vitro to further confirm the molecular mechanism of OGG1. Our findings shed novel insights on the molecular mechanism by which OGG1 influenced the development of pulmonary fibrosis.

## Methods

### Animals and interventions

Thirty-six male 6–8-week-old C57BL/6 mice weighing 18–22 g were purchased from Beijing Vital River Laboratory Animal Technology Co., Ltd. (Beijing, China), and were housed in a standard animal room with free access to water and food. All experimental procedures were performed in accordance with the National Institutes of Health Guide for the care and use of laboratory animals, and approved by Zhengzhou University Laboratory Animal Center.

To induce a pulmonary fibrotic mouse model, mice were received a single dose of 5 mg/kg BLM (Nippon Kayaku, Japan) using a Micro-Sprayer (Penn-Century, Wyndmoor, Philadelphia). The mice in Sham group were given the same amount of normal saline. One day after BLM induction, the pulmonary fibrosis mice were treated intraperitoneally with TH5487 (30 mg/kg/twice one week; OGG1-specific inhibitor, Selleck) in BLM + TH5487 group, and were treated intraperitoneally with TH5487 and Mitochondrial division inhibitor 1 (Mdivi-1; 10 mg/kg/twice one week; Selleck) in BLM + TH5487 + Mdivi-1 group. On the 28st day, all mice were euthanized with a lethal dose of sodium pentobarbital (50 mg/kg).

### Histological and immunofluorescent analysis

The left lung was fixed with 4% paraformaldehyde for 24 h, embedded with paraffin and then cut into 5-μm-thickness slices, followed by staining with haematoxylin and eosin (HE) solution (Beyotime, Shanghai, China) and Masson’s trichrome (Beyotime), respectively, to obverse the histopathological changes under a light microscopy (Olympus Corporation, Tokyo, Japan). Meanwhile, the lung tissues were also assessed by immunofluorescence assay. after deparaffinization, rehydration and antigen retrieval, the slices were blocked with 3% BSA and then probed with primary antibodies against CD86 (13395-1-AP, Proteintech, Wuhan, China), CD206 (18704-1-AP, Proteintech), CD68 (28058-1-AP, Proteintech), OGG1 (15125-1-AP, Proteintech), PINK1 (23274-1-AP), Vimentin (10366-1-AP, Proteintech), and LC3B (ab192890, Abcam, Cambridge, UK) at 4 °C overnight and then incubated with Alexa Fluor 488- and 594- conjugated secondary antibodies (ab150081 and ab150080, Abcam). The nuclei were stained with DAPI (Beyotime). The fluorescent images were captured under a fluorescence microscopy (Olympus Corporation).

### Western blot

Total proteins were isolated from tissue homogenates or cells using RIPA lysis buffer. After determining the protein concentration using a BCA Protein Assay Kit (Pierce, Rockford, IL), equal amounts of proteins (30 µg) were subjected to 12% SDS-PAGE and subsequently transferred onto PVDF membranes (Millipore, MA, USA). After blocked with nonfat milk, the membranes were probed with primary antibodies against Collagen I (14695-1-AP, Proteintech), α-SMA (14395-1-AP, Proteintech), PINK1 (23274-1-AP, Proteintech), Parkin (14060-1-AP, Proteintech), OGG1 (15125–1-AP, Proteintech), and GAPDH (10494-1-AP, Proteintech) at 4 °C overnight and then incubated with the HRP-conjugated goat anti-rabbit IgG secondary antibody (SA00001-2, Proteintech) at room temperature for 2 h. Finally, the blots were developed with an enhanced chemiluminescence kit (ECL; Amersham Pharmacia Biotech, Amersham, UK) and quantified with ImageJ software (National Institutes of Health, USA).

### Measurement of 4-hydroxynonenal (4-HNE), malondialdehyde (MDA), intracellular ROS and mitochondrial ROS (mtROS)

The 4-HNE level in the lung tissue homogenates was examined using the Lipid Peroxidation (4-HNE) Assay Kit (Abcam), and the MDA level was examined using its commercial kit from Jiancheng Bioengineering Institute (Nanjing, China), strictly in line with the manufacturer’s instructions, respectively. The intracellular ROS level was assessed adopting its commercial kit from Shanghai Jianglai Biological Technology Co., Ltd. (China).

For mtROS production, freshly prepared lung OCT sections were incubated with dihydroethidium (DHE; Sigma-Aldrich, USA) for 30 min in the darkness according to the manufacturer’s instructions. The images were captured under a fluorescence microscopy (Olympus Corporation).

### Cell culture and transfection

Human monocytic leukemia THP-1 cells and human lung fibroblast cells (HFL-1) were acquired from American Type Culture Collection (ATCC; USA) and cultured at 37 °C and 5% CO_2_ in a humidified incubator as we previously reported (Wu et al. 2022). THP-1 cells were differentiated to M0 macrophages by incubation with 150 ng/ml phorbol 12-myristate 13-acetate (PMA; Sigma-Aldrich), followed by induction with 10 ng/mL IL-4 (PeproTech, Israel) for the macrophage polarization to M2 phenotype. Subsequently, the conditional medium from macrophages were harvested and added to HFL-1 cells for culture.

For cell transfection, the full length of PINK1 was cloned into pcDNA 3.1 vector to construct the PINK1-overexpressing vector (OV-PINK1; GenePharma, Shanghai, China), with the empty pcDNA 3.1 vector as negative control (Ov-NC). The small interfere RNAs (siRNAs) targeting OGG1(si-OGG1#1, 5′-GCGCAAGUACUUCCAGCUATT-3′ and si-OGG1#2, 5′-GCGCAAGTACTTCCAGCTAGA-3′) were constructed. The scramble siRNA was used as the negative control (si-NC, 5′- CCGGCAACAAGATGAAGAGCACCAACTCGAGTTGGTGCTCTTCATCTTGTTGTTTTTG-3′). HFL-1 cells were transfected with above vectors adopting Lipofectamine 3000 reagent (Invitrogen, CA, USA) in line with the manufacturer’s guidelines. 48 h post transfection, cells were harvested for subsequent experiments.

### Measurement of intracellular ATP level

HFL-1 cells lysates were harvested and centrifugated at 12, 000 g for 5 min. The supernatant was added to ATP assay mix working solution, and the intracellular ATP content was measured using an ATP Detection kit (Beyotime) according to the manufacturer’s guidelines.

### Measurement of mitochondrial membrane potential (MMP)

The MMP was assessed using fluorescent JC-1 probe (Beyotime) in line with the manufacturer’s guidelines, where the JC-1 aggregates showed red fluorescence under a high MMP while JC-1 monomer showed green fluorescence under low MMP. The fluorescent images were captured under a fluorescent microscopy (Olympus Corporation) at emission of 590/530 nm with excitation of 525/490 nm.

### Enzyme-linked immunosorbent assay (ELISA)

The concentrations of Collagen I, α-SMA, TGF-β in the culture medium were examined adopting their corresponding ELISA kits (Cusabio Biotech, Wuhan, China) strictly in line with the manufacturer’s protocol.

### Statistical analysis

All data were presented as mean ± standard deviation (SD). All statistical analyses were performed using GraphPad Prism software (ver. 8.0; GraphPad, La Jolla, CA, USA). Student’s t-test or one way analysis of variance (ANOVA) were adopted for intergroup comparison, and the difference was statistically significant when P < 0.05.

## Results

### Macrophages polarization and mitophagy following pulmonary fibrosis

**Fig. 1:**
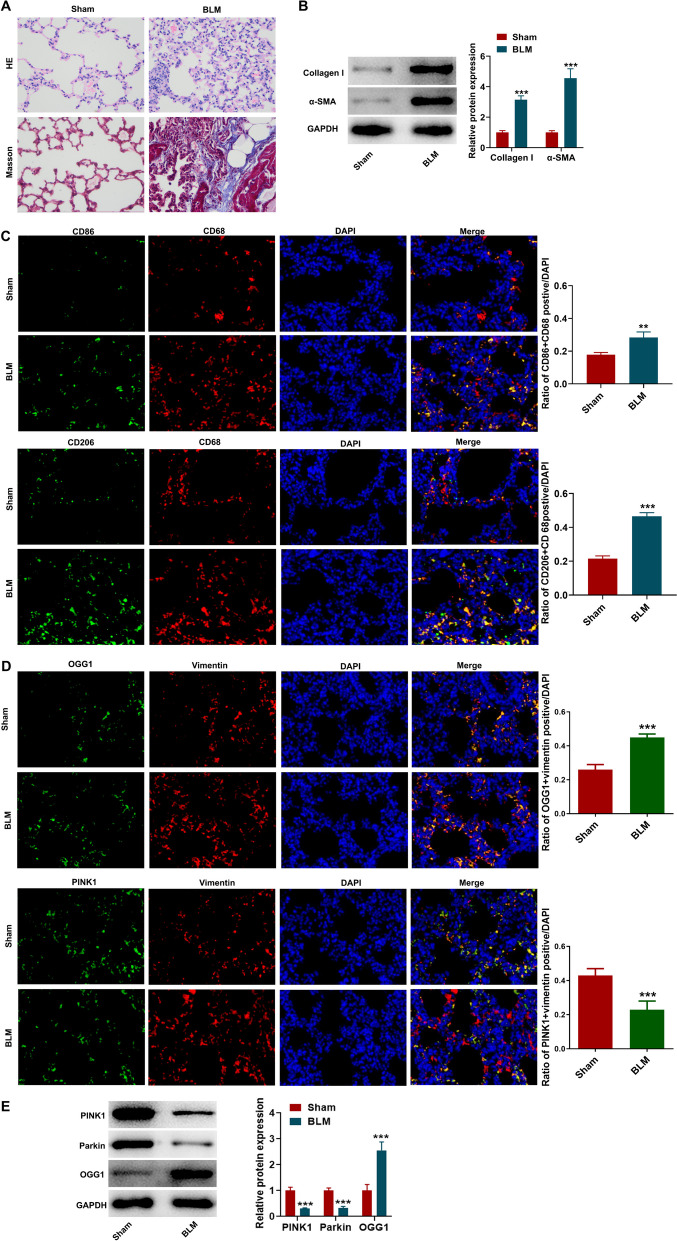
Macrophages polarization and mitophagy following pulmonary fibrosis. **A** BLM-induced pulmonary fibrosis animal model was established. HE staining and Masson staining were performed to observe the histological changes. **B** The expression level of Collagen I and α-SMA in lung tissues was examined by western blot. **C** Immunofluorescent analysis was used to detect CD68^+^CD86^+^ positive cells and CD68^+^CD206^+^ positive cells in lung tissues. **D** Immunofluorescent analysis was used to assess OGG1^+^Vimentin^+^ and PINK1^+^Vimentin^+^ positive cells in lung tissues. **E** The expression level of OGG1, PINK1 and Parkin in lung tissues was examined by western blot. **p < 0.01, ***p < 0.001

At first, BLM-induced pulmonary fibrosis animal model was established. As shown in Fig. 1A that compared to the Sham, obvious damage of the lung structure and the excessive deposition of collagen were observed in BLM group. Meanwhile, the protein expression of Collagen I and α-SMA, the classical hallmark of fibrosis, was validated to be upregulated in lung tissues after BLM induction (Fig. 1B). According to immunofluorescence assay, the CD68^+^CD86^+^ positive (M1) cells and CD68^+^CD206^+^ positive (M2) cells were elevated following BLM induction, and especially CD68^+^CD206^+^ positive cells exhibited a dramatically dominant increase (Fig. 1C), revealing that pulmonary fibrosis triggered macrophages activation, and M2 phenotype macrophages occupied dominantly. In addition, compared to Sham group, the OGG^+^Vimentin^+^ positive cells in lung tissues were remarkably elevated while the the PINK1^+^Vimentin^+^ positive cells were remarkably decreased in BLM group (Fig. 1D). Considering Vimentin as a fibroblast-specific marker, the findings confirmed the upregulated OGG1 expression and downregulated PINK1 expression in fibroblasts of BLM-induced mice. Consistently, the western blot assay revealed that protein expression of OGG1 was significantly increased in BLM group, while the protein expression of PINK1 and Parkin was significantly decreased (Fig. 1E), suggesting that OGG1/PINK1 mitophagy might be involved in the progression of pulmonary fibrosis.

### TH5487 mitigates BLM-induced pulmonary fibrosis and M2 macrophage polarization, which is partly hindered by Mdivi-1

**Fig. 2:**
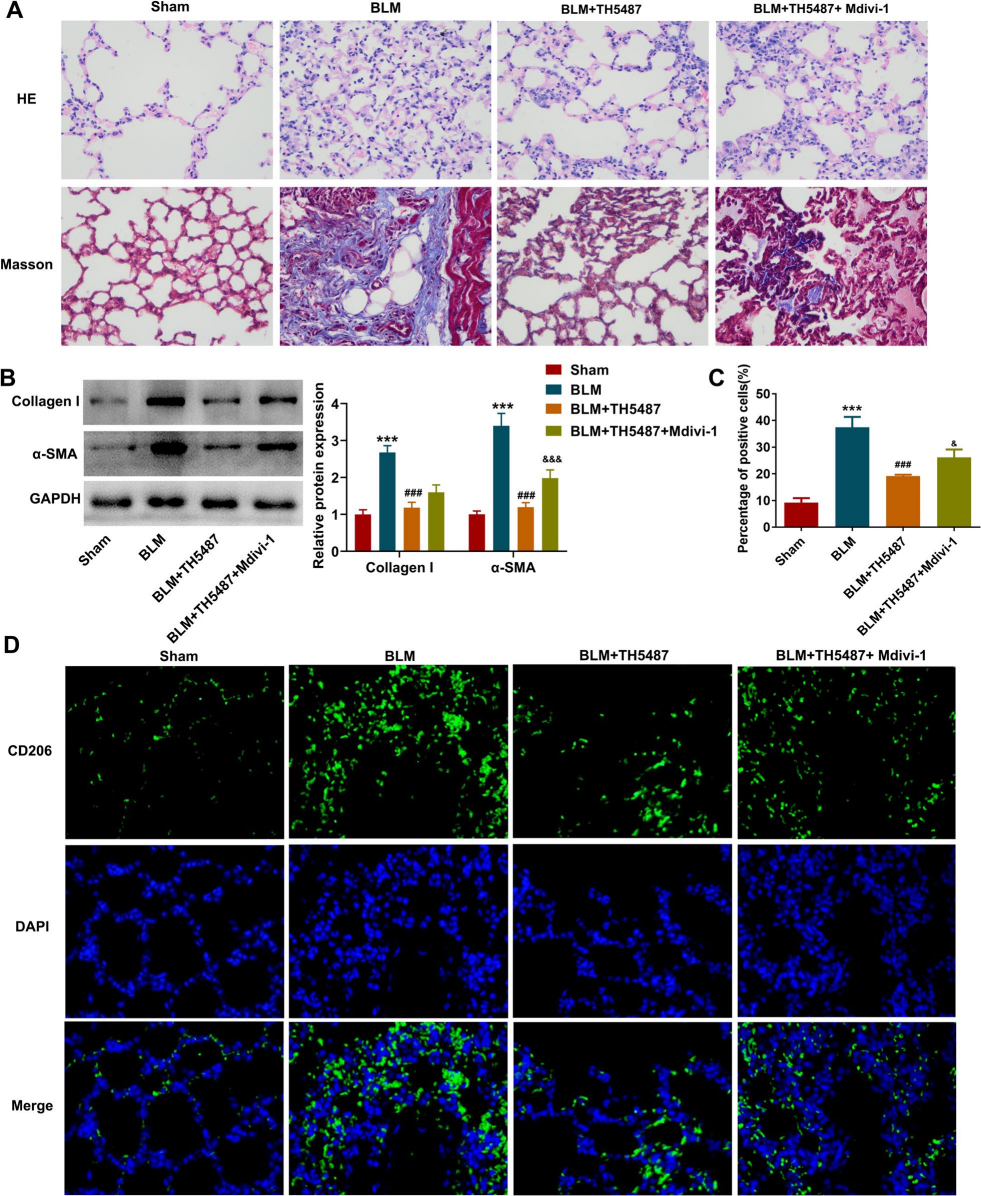
TH5487 mitigates BLM-induced pulmonary fibrosis and M2 macrophage polarization, which is partly hindered by Mdivi-1. **A** BLM-administrated mice were treated with TH5487 or co-treated with TH5487 and Mdivi-1. HE staining and Masson staining were performed to observe the histological changes. **B** The expression level of Collagen I and α-SMA in lung tissues was examined by western blot. **C**-**D** Immunofluorescent analysis was used to detect CD206-positive (M2) cells. ***p < 0.001 vs Sham; ###p < 0.001 vs BLM; &p < 0.05, &&&p < 0.001 vs BLM + TH5487

To clarify whether OGG1 participated into the progression of pulmonary fibrosis through regulating mitophagy, BLM-administrated mice were treated with TH5487 or co-treated with TH5487 and Mdivi-1. As shown in Fig. 2A, B that the damaged lung structure and the excessive collagen deposition following BLM induction were partly attenuated by TH5187 treatment, and the BLM-elevated Collagen I and α-SMA expression was also reduced following TH5187 treatment, demonstrating that TH5487 administration alleviated pulmonary fibrosis. Wherever, the relief caused by TH5187 treatment was partly abolished by additional treatment with Mdivi-1, indicating that inhibition of OGG1 might relieve pulmonary fibrosis partly through activating mitophagy. Meanwhile, TH5487 exerted an inhibitory effect on BLM-elevated CD206 expression, which was partly restricted by Mdivi-1 (Fig. 2C, D), indicating that inhibition of OGG1 could retard M2 macrophage polarization following pulmonary fibrosis partly through activating mitophagy.

### TH5487 mitigates BLM-induced oxidative stress and mitophagy of mice, which is partly hindered by Mdivi-1

**Fig. 3:**
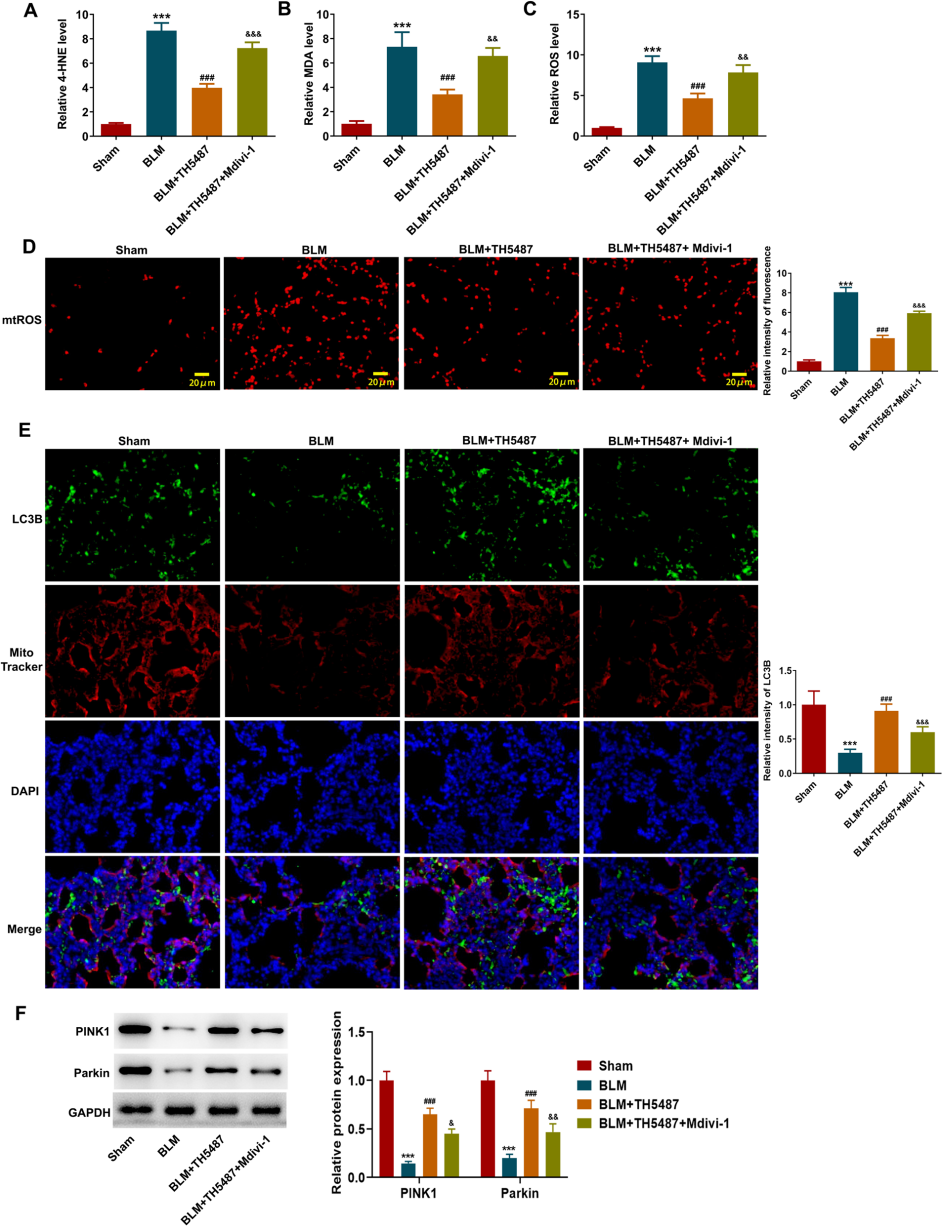
TH5487 mitigates BLM-induced oxidative stress and mitophagy of mice, which is partly hindered by Mdivi-1. **A**-**C** BLM-administrated mice were treated with TH5487 or co-treated with TH5487 and Mdivi-1. The levels of 4-NHE, MDA and ROS of lung tissues was examined using their corresponding commercial kits. **D** Representative images of mtROS production in lung OCT sections. **E** Representative images of LC3B-MitoTracker double immunofluorescent staining of lung tissues. **F** The expression level of PINK1 and Parkin in lung tissues was examined by western blot. ***p < 0.001 vs Sham; ###p < 0.001 vs BLM; &p < 0.05, &&p < 0.01, &&&p < 0.001 vs BLM + TH5487

In addition, it was observed from Fig. 3A–C that compared to Sham group, the levels of 4-NHE, MDA and ROS were greatly elevated in BLM group, while TH5487 administration exerted an antioxidant activity through declining 4-NHE, MDA and ROS levels, which was partly abolished by Mdivi-1. Meanwhile, mtROS was also excessively produced following pulmonary fibrosis, which was partly blocked by TH5487 treatment while was facilitated by additional treatment with Mdivi-1 (Fig. 3D). Moreover, mitophagy was measured via co-location of MitoTracker (a mitochondrial marker) and LC3B (an autophagosome marker) double fluorescence staining in lung tissues, and the results showed that the mitophagy is substantially reduced in BLM group while was markedly increased following TH5487 administration, which was partly reversed by additional Mdivi-1 treatment (Fig. 3E). Further, BLM-induced low expressions of PINK1 and Parkin were significantly upregulated following TH5487, which was partly abolished by additional Mdivi-1 treatment (Fig. 3F). Taken together, these findings confirmed that TH5487 treatment effectively reduced oxidative stress, promoted PINK1/Parkin-mediated mitophagy and attenuated mitochondrial dysfunction in BLM-induced pulmonary fibrosis mice.

### PINK1 overexpression restricts M2 macrophages-induced mitochondrial dysfunction and mitophagy inactivation in lung fibroblast cells

**Fig. 4:**
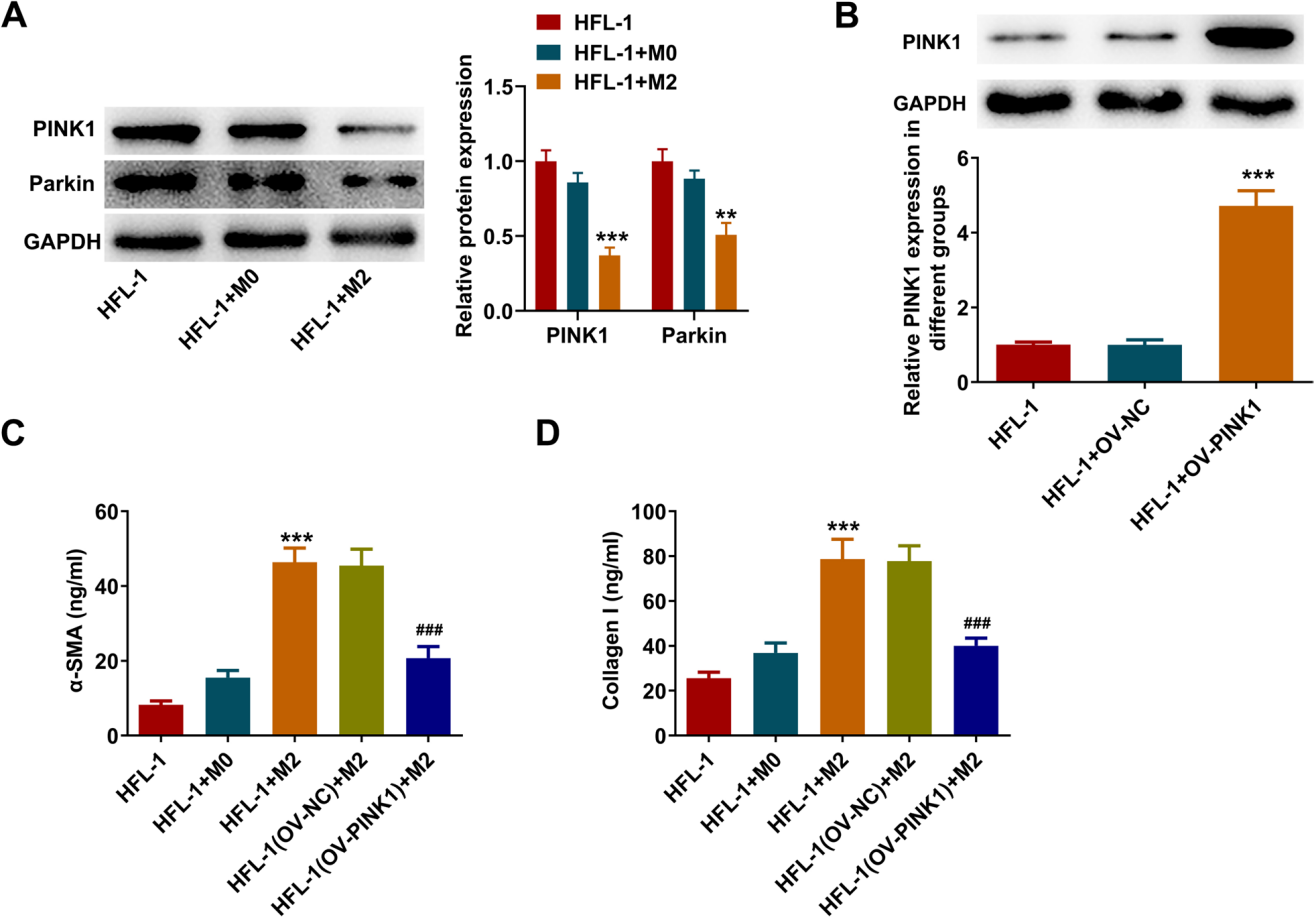
PINK1/Parkin in M2 macrophages-cultured HFL-1 cells. **A** The conditional medium from M0/M2 macrophages was harvested and added to HFL-1 cells for culture, and the protein expression of PINK1 and Parkin in HFL-1 cells was examined by western blot. **p < 0.01, ***p < 0.001 vs HFL-1. **B** HFL-1 cells were received transfection, and the protein expression of PINK1 was examined by western blot. ***p < 0.001 vs HFL-1 + Ov-NC. **C**-**D** HFL-1 cells or PINK1-overexpressing HFL-1 cells were cultured in the conditional medium from macrophages, and the Collagen I and α-SMA levels in HFL-1 cells were examined by ELISA assay. ***p < 0.001 vs HFL-1; ###p < 0.001 vs HFL-1(OV-NC) + M2

Next, to further confirm the molecular regulation of OGG1/PINK1 during pulmonary fibrosis, the loss- and gain-of function experiments were conducted in vitro. At first, as observed from in vivo findings, macrophage polarization toward M2 phenotype is a critically variable factor for pulmonary fibrosis progression, hence the conditional medium from M2 macrophages was harvested and added to HFL-1 cells for culture, so as to simulate the immune microenvironment around fibroblasts during pulmonary fibrosis. It was observed from Fig. 4A that after culturing with the conditional medium from M2 macrophages, the protein expression of PINK1 and Parkin in HFL-1 cells was greatly decreased. Considering the critical role of PINK1/Parkin for driving mitophagy, M2 macrophages might partly contribute to the inactivation of mitophagy in fibroblasts. Subsequently, HFL-1 cells were transfected with OV-PINK1 to over-express PINK1 (Fig. 4B), and then the PINK1-overexpressing HFL-1 cells were cultured in the conditional medium from macrophages, and the results in Fig. 4C, D revealed that PINK1 overexpression could significantly inhibit M2 macrophages-induced high Collagen I and α-SMA levels in HFL-1 cells.

**Fig. 5:**
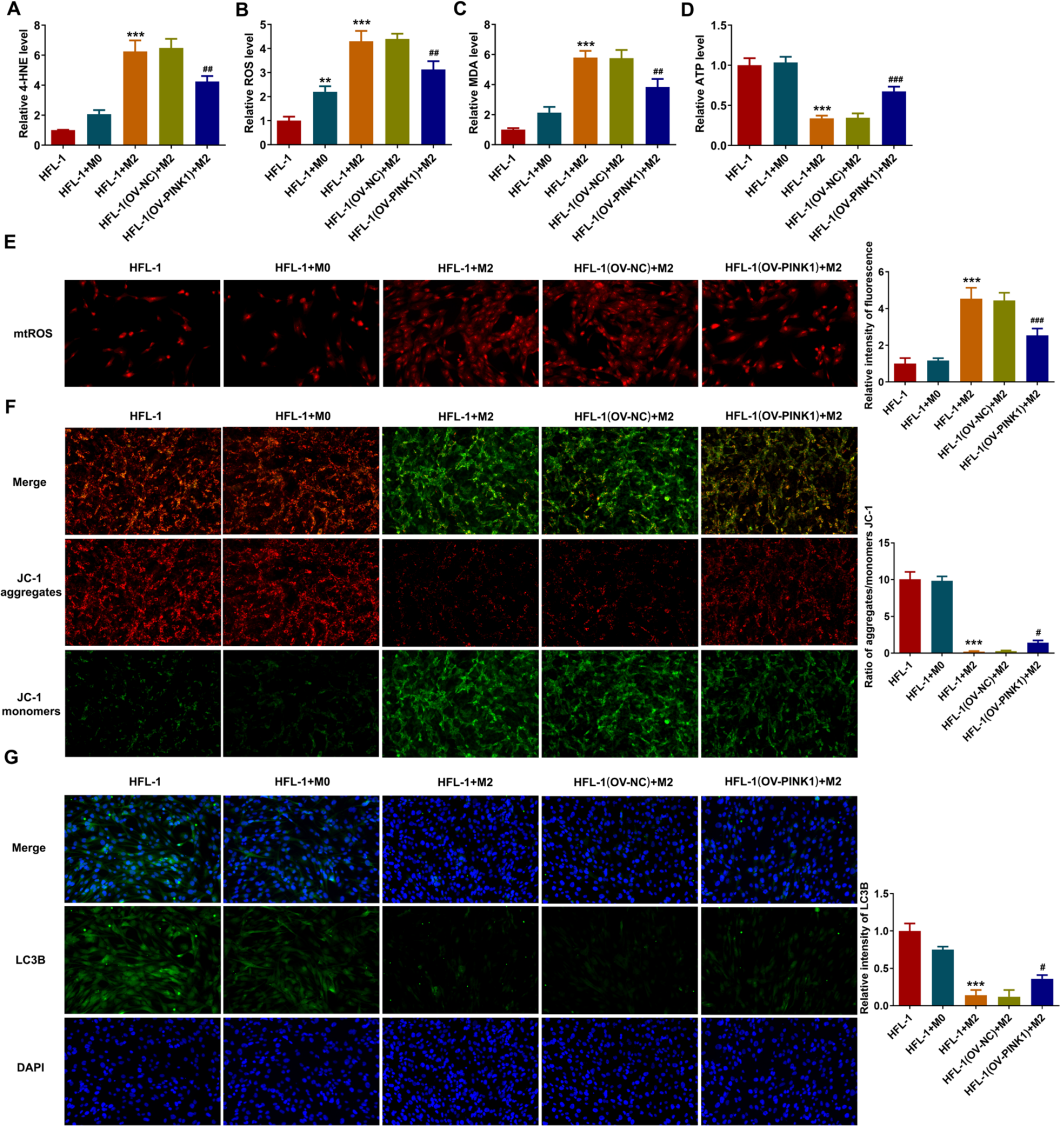
PINK1 overexpression restricts M2 macrophages-induced mitochondrial dysfunction and mitophagy inactivation in lung fibroblast cells. **A**-**D** The levels of 4-NHE, MDA, intracellular ROS and ATP in HFL-1 cells were examined using their corresponding commercial kits. **E** Representative images of mtROS production in HFL-1 cells. **F** The MMP level of HFL-1 cells was examined by JC-1 staining. **G** Representative images of LC3B immunofluorescent intensity of HFL-1 cells. **p < 0.01, ***p < 0.001 vs HFL-1; #p < 0.05, ##p < 0.01, ###p < 0.001 vs HFL-1(OV-NC) + M2

In addition, PINK1 overexpression also significantly inhibited excessive production of 4-HNE, MDA, intracellular ROS and mtROS, and enhanced ATP level in HFL-1 cells cultured with conditional medium from M2 macrophages (Fig. 5A–E). The fluorescent images from JC-1 staining disclosed that M2 macrophages markedly reduced MMP of HFL-1 cells, which partly abolished by PINK1 overexpression (Fig. 5F). Moreover, the reduced LC3B expression of HFL-1 cells caused by M2 macrophages was partly elevated after PINK1 overexpression (Fig. 5G). Taken together, above findings suggested that enhancing PINK1-mediated mitophagy effectively attenuated M2 macrophages-caused oxidative stress and mitochondrial dysfunction in fibroblasts.

### OGG1 knockdown inhibits M2 macrophages-induced mitochondrial dysfunction in lung fibroblast cells

**Fig. 6:**
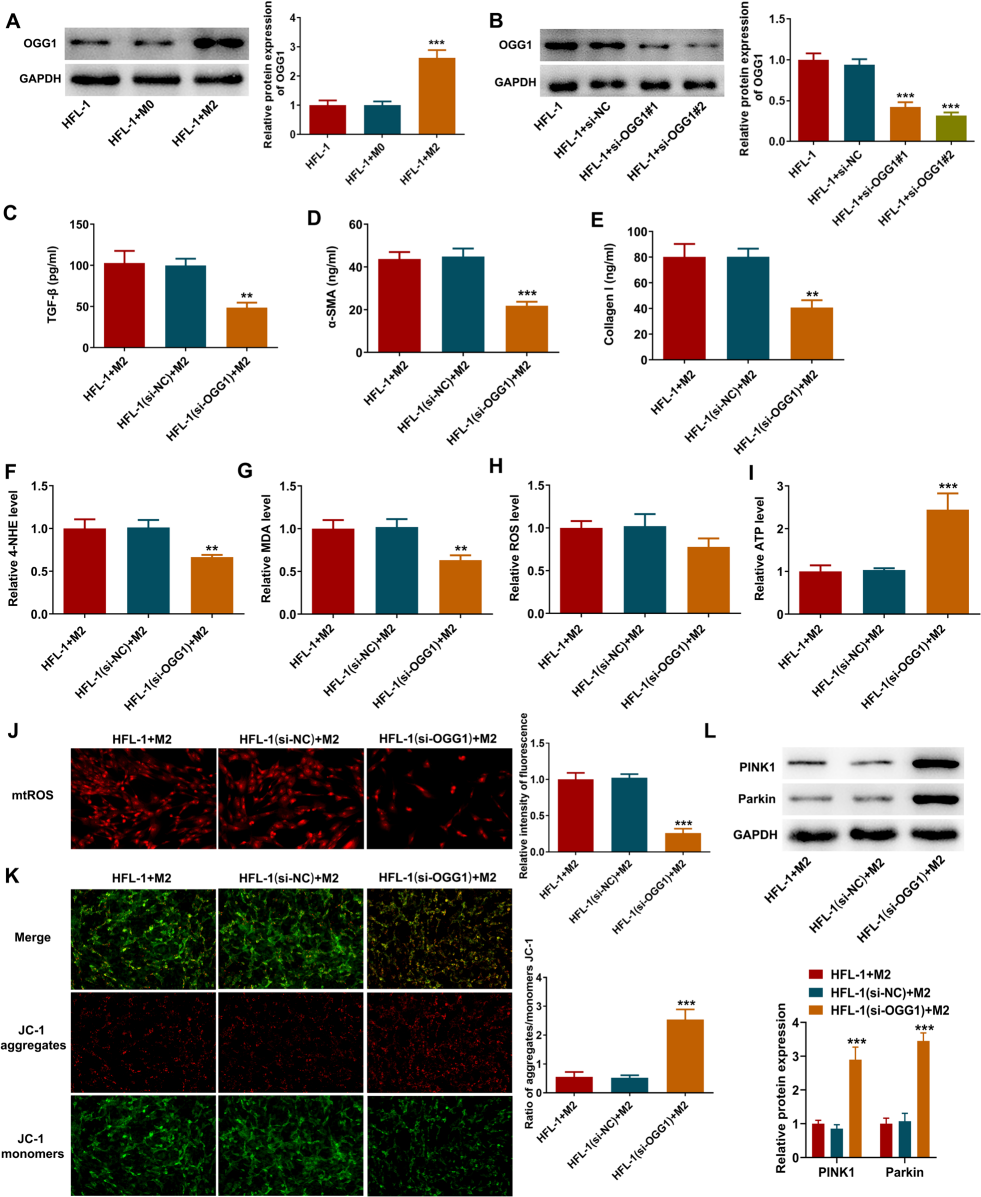
OGG1 knockdown inhibits M2 macrophages-induced mitochondrial dysfunction in lung fibroblast cells. **A** The conditional medium from M0/M2 macrophages was harvested and added to HFL-1 cells for culture, and the protein expression of OGG1 in HFL-1 cells was examined by western blot. ***p < 0.001 vs HFL-1. **B** HFL-1 cells were received transfection, and the protein expression of OGG1was examined by western blot. ***p < 0.001 vs HFL-1 + si-NC. **C**-**E** HFL-1 cells or OGG1-knockdown HFL-1 cells were cultured in the conditional medium from M2 macrophages, and the TGF-β, Collagen I and α-SMA levels in HFL-1 cells were examined by ELISA assay. **F**-**I** The levels of 4-NHE, MDA, intracellular ROS and ATP in HFL-1 cells were examined using their corresponding commercial kits. **J** Representative images of mtROS production in HFL-1 cells. **K** The MMP level of HFL-1 cells was examined by JC-1 staining. **L** The protein expression level of PINK1 and Parkin in HFL-1 cells was examined by western blot. **p < 0.01, ***p < 0.001 vs HFL-1(si-NC) + M2

Furthermore, after cultured with the conditional medium from M2 macrophages, the expression level of OGG1 in HFL-1 cells was significantly elevated (Fig. 6A), consistent with our previous findings in vivo. Subsequently, to validate the regulatory mechanism of OGG1 in vitro, the loss-of-function experiments were conducted. It was observed from Fig. 6B that compared to HFL-1 + si-NC group, the expression level of OGG1 was significantly decreased in HFL-1 + si-OGG1#1 or HFL-1 + si-OGG1#2 group. Attributed to a superior transfection efficacy, si-OGG1#2 was used for following experiments. Thereafter, it was found that OGG1 knockdown not only reduced TGF-β, Collagen I and α-SMA concentrations, but also inhibited 4-HNE, MDA and mtROS production, while promoted ATP level in HFL-1 cells cultured with the conditional medium from M2 macrophages (Fig. 6C–J). Meanwhile, according to the JC-1 staining, OGG1 knockdown elevated the MMP level of HFL-1 cells (Fig. 6K). Additionally, the protein expression level of PINK1 and Parkin in HFL-1 cells was greatly promoted following OGG1 knockdown (Fig. 6L).

## Discussion

Pulmonary fibrosis is a chronic progressive interstitial lung disease and is one of the major causes for deaths around the world. The current drugs can effectively delay the progression of lung failure but still fail to cure pulmonary fibrosis completely (Somogyi 2019). Hence novel approaches and therapeutic targets are required to be developed. Previous studies have highlighted the protective role of OGG1 inhibition against pulmonary fibrosis in animal model; however, the specific mechanism of action for its regulatory role has been not clarified yet (Tanner et al. 2023; Ling et al. 2022). In the present study, we made a further exploration on the regulatory mechanism of OGG1 impacting the progression of pulmonary fibrosis, which was closely associated with macrophages polarization and mitophagy.

Macrophages, the most abundant immune cells in the lungs, are crucially important sentinels integral to host pulmonary defense (Patel and Metcalf 2018; Li et al. 2019; Zhang et al. 2018). In response to stimulation or signals, macrophages are generally activated and exhibit two distinctive functional phenotypes: M1-phenotype macrophages, the pro-inflammatory phenotype characterized by expression of molecules such as CD86, as well as the production of a series of pro-inflammatory cytokines such as interleukin-1β (IL-1β) and tumor necrosis factor (TNF-α); M2-phenotype macrophages, the anti-inflammatory phenotype hallmarked by CD206, accompanied with anti-inflammatory mediators (Murray 2017). It was notable that fibroblast activation and proliferation is caused by activated M2 macrophages which produce profibrotic factor TGF-β to facilitate the secretion of collagen and extracellular matrix proteins and thereby contributing to pulmonary fibrosis (Misharin et al. 2017). Therefore, the predominant infiltration of M2 macrophages in lung tissues serve as a crucial mediator of fibrogenesis, and reduction of M2 macrophages has been considered as an alternative approach to alleviate pulmonary fibrosis (Zhang et al. 2018; Cheng et al. 2021). For instance, eucalyptol was suggested as a potential therapeutic agent for pulmonary fibrosis through disturbing M2 macrophage polarization and reducing macrophages-mediated production of TGF-β (Rui et al. 2022). Meanwhile, silencing of Methyl-CpG Binding Domain Protein 2 (MBD2) could significantly prevent mice from BLM-mediated pulmonary fibrosis via inhibiting macrophage M2 program, and thus MBD2 was regarded as a viable target against pulmonary fibrosis (Wang, et al. 2021). In agreement with previous studies, our findings revealed that OGG1 expression was elevated following BLM-induced pulmonary fibrosis, accompanied with elevated M2 macrophages. TH5487 administration not only attenuated pro-fibrotic phenotypes of BLM-induced mice, but also reduced M2 macrophages. Therefore, TH5487-reduced M2 macrophages might partly account for the protective role of TH5487 against pulmonary fibrosis in vivo. Interestingly, it is well-recognized that the M2 macrophages polarization are induced by the T-helper-2 (Th2) cytokines IL-4 and IL-13 which are elevated in several lung diseases involving fibrosis, and TH5487 has been demonstrated to cause a considerable decrease of IL-4 and IL-13, as well as reduced macrophages, in asthma mice (Tanner et al. 2022). Accordingly, it is possible that TH5487 can also target other cells, and the reduced M2 macrophages in TH5487-treated mice may be also related to Th2 cells.

In addition, attributed to the importance of M2 macrophages for fibrogenesis, the conditional medium from M2 macrophages was employed to culture HFL-1 cells, so as to simulate the immune microenvironment around fibroblasts during pulmonary fibrosis. The in vitro findings showed that conditional medium from M2 macrophages could facilitate the production of profibrotic proteins in fibroblasts, which was significantly restricted by OGG1-depleted HFL-1 cells, suggesting that OGG1 inhibition could directly relieve pro-fibrotic factors in fibroblasts, which was consistent with the protective role of TH5487 against pulmonary fibrosis in vivo.

PINK1/Parkin is currently the most widely studied pathway involved in the elimination of damaged mitophagy in mammals (Lu et al. 2023). Generally speaking, when mitochondria are damaged following external simulation, the damaged mitochondria will depolarize and the MMP will be lost. Subsequently, PINK1 stably accumulates in the outer membrane of impaired mitochondria to activate Parkin, thereby promoting the degradation of damaged mitochondria via recruitment of the autophagy adaptor and microtubule-associated protein LC3 and ultimately leading to a restoration of dysfunctional mitochondria (Lu et al. 2023; Bhatia, et al. 2019; Bingol and Sheng 2016). Nowadays, mitophagy plays an important role in human disease and hence serves as a therapeutic target for the treatment of mitochondrial diseases including pulmonary fibrosis (Dombi et al. 2018). The deficiency of PINK1 or Parkin has been demonstrated to impair mitochondrial homeostasis and promote lung fibrosis (Bueno et al. 2015; Patel et al. 2015), highlighting the importance of PINK1/Parkin-mediated mitophagy during pulmonary fibrosis. Accumulating evidence has confirmed the abnormal expression and the regulatory role of PINK1 in type II alveolar epithelial cells and lung tissues following pulmonary fibrosis (Bueno et al. 2019), while PINK1/Parkin-mediated mitophagy in in vitro fibroblasts is rarely explored. In this study, we not only demonstrated that inhibition of PINK1/Parkin/mitophagy partly weakened the protective effect of OGG1 inhibition against BLM-induced pulmonary fibrosis in vivo, but also disclosed that PINK1 overexpression effectively attenuated M2 macrophages-mediated oxidative stress, mitochondrial dysfunction in fibroblasts in vitro.

Of note, a latest article revealed that OGG1 could inhibit mitophagy by binding to PINK1 and regulating the transfer of PINK1 into mitochondria to aggravate renal ischemia–reperfusion injury, highlighting the importance of OGG1/PINK1/mitophagy axis in modulating pathological processes (Zhao et al. 2023). Consistently, in the present study, we found that OGG1 could negatively regulate PINK1/Parkin expression during pulmonary fibrosis both in vivo and in vitro, providing evidence that OGG1/PINK1/mitophagy axis acted as a critical mediator against pulmonary fibrosis through. Further, our findings revealed that OGG1^+^ fibroblasts were clearly increased and Mdivi-1 rescued the number of M2 macrophages that TH5487 decreased in vivo, suggesting that mitophagy in fibroblasts might affect M2 polarization and form feedback-loop between M2 macrophages and fibroblasts, even if the contribution might be small. Taken together, OGG1/PINK1/mitophagy axis in fibroblasts played a crucial role in regulating pulmonary fibrosis and the relevant macrophage polarization.

However, several limitations exist in our study. Firstly, our findings showed that TH5487 attenuated M2 polarization in vivo, but we did not check whether TH5487 could directly inhibit M2 macrophage differentiation in vitro. Secondly, TH5487 administration in mice is not a targeted drug delivery, and it may affect multiple cells in vivo, hence whether TH5487 can also target other cells to attenuate pulmonary fibrosis is deserved to be explored in our future work.

## Conclusion

In summary, this study provides substantial evidence indicating the involvement of OGG1 during pulmonary fibrosis and alleviation of pulmonary fibrosis by OGG1 inhibition. The protective role of OGG1 inhibition against pulmonary fibrosis is archived partly via activating PINK1/Parkin mitophagy and retarding M2 macrophage polarization. Therefore, OGG1 appears to be a promising clinical target with therapeutic potential for pulmonary fibrosis.

## Data Availability

All data generated in this study have been included in this article.
